# Exosomal cyclophilin A as a novel noninvasive biomarker for Epstein‐Barr virus associated nasopharyngeal carcinoma

**DOI:** 10.1002/cam4.2185

**Published:** 2019-05-07

**Authors:** Lingzhi Liu, Lielian Zuo, Jing Yang, Shuyu Xin, Jing Zhang, Jianhua Zhou, Guiyuan Li, Jinyong Tang, Jianhong Lu

**Affiliations:** ^1^ NHC Key Laboratory of Carcinogenesis, Department of Pathology Xiangya Hospital, Central South University Changsha China; ^2^ Department of Microbiology, Cancer Research Institute School of Basic Medical Science, Central South University Changsha China; ^3^ Department of Otolaryngology‐Head and Neck Surgery The First People's Hospital of Chenzhou Hunan China

**Keywords:** Cyclophilin A, EBV‐VCA‐IgA, Epstein‐Barr virus, exosomes, nasopharyngeal carcinoma

## Abstract

Exosomes have emerged as novel vehicles for proteins and other contents in cancer progression. Cyclophilin A (CYPA) is a pivotal member of immunophilin family. Whether CYPA can be detected in sera of nasopharyngeal carcinoma (NPC) patients remains to be explored. Epstein‐Barr virus (EBV) is the first identified human tumor virus and is a causative agent of NPC. The antibody of EBV capsid antigen immunoglobulin A (EBV‐VCA‐IgA) is a known biomarker of NPC, with a proportion of no more than 70% being detected positively. Hence, novel biomarkers need to be discovered for early diagnosis, prognosis, and monitoring of EBV‐associated NPC. A total of 110 NPC and 36 normal control serum samples were collected. Exosomes from these samples were extracted. The mRNA and protein expression levels of the above samples were validated by reverse transcription –quantitative polymerase chain reaction, Western blotting, or enzyme‐linked immunosorbent assay (ELISA). Finally, the results demonstrated that both the serum and exosomal CYPA levels of NPC patients were significantly higher than that of normal cases. In addition, exosomal CYPA had a much higher level than that in the whole sera. The positive rate of EBV‐VCA‐IgA antibody was 68.2% in NPC sera, and noticeably, among the cases with EBV‐VCA‐IgA negative, 80% of them presented high levels of CYPA above the standard (cutoff value). In particular, CYPA in exosomes was uniformly with higher significance than that in whole sera. Combined analysis of CYPA protein and EBV‐VCA‐IgA antibody showed a greatly higher discriminatory ability in diagnosis of NPC. Moreover, exosomal CYPA level had a positive correlation with that of the EBV‐encoded latent membrane protein 1 (LMP1) in exosomes. EBV‐positive cancer cells secreted significantly higher levels of exosomal CYPA. This study established the utility of circulating exosomal CYPA as a potential noninvasive diagnostic biomarker for EBV‐associated NPC.

**Abbreviations**
AUC: areas under the ROC curve
CsA: cyclosporine A
CYPA: cyclophilin A
EA: intracellular early antigen
EBV: Epstein‐Barr virus
EBV‐VCA‐IgA: IgA antibodies against EBV capsid antigen
ELISA: enzyme‐linked immunosorbent assay
HRP: Horseradish peroxidase
LMP1: EBV latent membrane protein 1
NPC: nasopharyngeal carcinoma
PBS: phosphate‐buffered saline
PPlase: pepidyl prolylcis‐trans isomerase activity
qPCR: quantitative real‐time polymerase chain reaction
ROC: receiver operating characteristics

## 1 INTRODUCTION

Nasopharyngeal carcinoma (NPC), especially the undifferentiated subtype, is epidemic in southern China.^1^ Advances in diagnostic techniques and the systemic therapy of radiotherapy, chemotherapy, and other technologies have been put into application, thus making the incidence and mortality from this malignancy decrease substantially over the past decades. It has been shown that age‐standardized incidence rates (1970‐2007) of NPC reduced significantly in epidemic areas by −0.9%‐−5.4% in males and −1.1%‐−4.1% in females every year in average, owing to lifestyle changes and economic development and another study revealed that age‐standardized mortality rates (1970‐2013) declined varying from −0.9% to 3.7% and −0.8% to −6.5% in males and females, respectively. These changes are probably due to diagnostic accuracy and combination of diverse treatment approaches including the radiotherapy techniques.^2^ However, distant metastasis remains a major issue. Thus, the prevention and early detection of NPC are still major issues to be concerned.

Epstein‐Barr virus (EBV) is a ubiquitous human herpesvirus, and establishes lifelong latent infection with more than 90% seropositively in adults.^3^ So far, quantitative assessment of circulating EBV DNA has been recognized in clinics for population screening, disease surveillance, and prognostication as a potent biomarker in NPC.^4^, ^5^ Besides, immunoserological markers such as IgA antibodies against EBV capsid antigen (EBV‐VCA‐IgA), have shown significant clinical value and presented as standard methods for early diagnosis.^6^ However, the positive rate of EBV‐VCA‐IgA detection usually reaches only about 60%‐70% in NPC patients probably due to the immunocompromise in a proportion of patients. This cannot satisfy the clinical need. We tried to seek a protein that is derived from the tumor cells and might not be impacted by immune ability to replenish this defect. This lead to the detection of the protein cyclophilin A (CYPA) as described below in the present approach. The EBV latent membrane protein 1 (LMP1) is a latent oncogenic protein encoded by EBV. Studies show that LMP1 harbors the oncogenic potential through NF‐κB signaling pathway and contributes to the viral life cycle in differentiating epithelia.^3^, ^7^

CYPA is a member of the immunophilin family with peptidyl prolyl cis‐trans isomerase (PPIase) activity, which is in close relationship with many physiological and pathological activities including protein folding and trafficking.^8^, ^9^ CYPA has been shown to be associated with various diseases such as cardiovascular disease, kidney disease, viral infection and cancer by binding with membrane receptor or intracellular partners and activating downstream signaling pathways.^10^, ^11^, ^12^, ^13^, ^14^

CYPA is not only an intracellular protein, but also can be secreted out of cells. Extracellular CYPA also plays critical role in a number of cancers. It promotes carcinogenesis, tumor invasion, and drug resistance in various cancer cell types.^15^, ^16^ In our laboratory, Yang et al previously performed 2D‐DIGE combined with MALDI‐TOF‐MS analysis and found that CYPA expression was upregulated in NPC tissues from the early stage.^17^ Since NPC is located deeply in the nasal cavity, plasma, or serum liquid biopsy becomes an ideal noninvasive method to help the diagnosis. Whether CYPA become detectable in sera and how it acts as a potential serum biomarker in NPC remains to be explored.

Exosomes are of 50‐200 nm extracellular vesicles and play critical roles in intracellular communication. Exosomes contain DNA, RNA, proteins and other bioactive molecules, playing roles in exchanging genetic information and regulating physiological and pathological activities.^18^, ^19^ Emerging studies have proven that exosomes released from tumor cells can affect tumor formation, growth, angiogenesis, metastasis, drug resistance, and immune evasion.^20^, ^21^, ^22^, ^23^ Noninvasive methods and potential biomarkers are essential for early detection. In pathological state, body fluids could release exosomes that contain altered composition and aberrant expression of exosomal components, indicating that exosomes and its contents are promising to be used as novel cancer biomarkers and therapeutic targets. In this study, a comparative analysis was performed for the CYPA levels both in whole sera and serum exosomes, as well as the EBV‐VCA‐IgA antibody in NPC patients. The results implied the level of CYPA, especially in exosomes combined with EBV‐VCA‐IgA can be used to diagnose EBV‐associated NPC. In addition, since LMP1 plays a central role in the development of NPC and is able to be detected in exosomes,^24^, ^25^, ^26^ we detected the exosomal LMP1 which might be associated with exosomal CYPA. The results implied that exosomal CYPA could become a promising biomarker for EBV‐associated NPC.

## 2 MATERIALS AND METHODS

### 2.1 Gene Expression Omnibus data analysis

Gene expression levels of a total of 41 samples (accession number: GDS3341) were retrieved from the Gene Expression Omnibus (GEO) database (https://www.ncbi.nlm.nih.gov/geo/). The target protein expression data were analyzed using GraphPad Prism 5 software with normal cases (NC) (n = 10) and NPC (n = 31) samples.

### 2.2 Serum samples and tissue biopsies

Clinical serum samples and tissue samples were mainly obtained from the Second Hospital of Xiangya, Central South University, with informed consent from all the patients. The clinical tumor biopsies from 10 patients had been diagnosed as undifferentiated NPC carcinoma by pathologists and the biopsies from 5 normal cases were from healthy individuals at random. The sera from 110 NPC patients and 36 normal cases were isolated from the venous blood samples by centrifugation at 1000 × g at 4°C for 10 minutes and stored at −80°C.

### 2.3 Cell lines and culture

Tumor cells are kept in our laboratory that kindly provided by the pioneers that established those laboratories.^27^, ^28^, ^29^ C666‐1 is an EBV‐positive NPC cell line.^29^ HK‐1 and 5‐8F are EBV‐negative NPC cell lines. HK‐1, derived from well‐differentiated NPC tissue, is a nonmetastatic cell line, and 5‐8F is a highly metastatic one. AGS is a kind of EBV‐negative gastric cancer cell line, and AGS‐EBV is an EBV‐positive gastric cancer cell line. GES‐1, is derived from a normal gastric tissue. BJAB is an EBV‐negative human lymphoma cell line, and Raji is an EBV‐positive human lymphoma cell line. The human embryonic kidney HEK293 or 293 was ATCC origin (catalog number: CRL‐1573) and utilized for the establishment of the cell line, C2089 by being transfected with the whole EBV genome (p2089),^30^, ^31^, ^32^ as described by our group.^33^ All these cell lines are grown in RPMI‐1640 (Gibco, California, USA) or Dulbecco's modified Eagle' s medium (Gibco, California, USA) supplemented with 10% FBS (fetal bovine serum) and 5% of CO_2_ at 37°C.

### 2.4 Extraction of serum exosomes

Exosomal fraction from 100 microliter of serum was isolated by using the ExoQuick™ kit (System Biosciences Inc, Mountain view, CA, USA), according to the manufacturer's instructions. Briefly, 1/4 volume of ExoQuick Solution was added to serum samples and then the mixture was refrigerated at 4°C overnight. In the following day, the mixture was centrifuged at 1500 × *g* for 30 minutes and supernatant was removed by aspiration. Pelleted fraction was re‐suspended in nuclease‐free water or prepared for the next processing.

### 2.5 Extraction of cellular supernatant exosomes

Exosomes were isolated from the culture supernatant of cells. About 20 mL of each cellular supernatant was collected and centrifuged at 3000 × *g* for 15 minutes at 4°C to remove cellular debris. The supernatant was mixed with 4 mL of ExoQuick–TC solution (System Biosciences Inc, Mountain view, CA, USA) and incubated at 4°C overnight. The mixture was then centrifuged at 10,000 × *g* for 15 minutes at 4°C, and the precipitates were obtained to be used for subsequent experiments.

### 2.6 Electron microscopy

Exosomes were spotted onto formvar‐carbon‐coated grids (200 mesh) and fixed with 2% (wt/vol) of paraformaldehyde for 5 minutes at room temperature. Then uranyl acetate was used to stain the exosomes. The grids were visualized under an FEI Tecnai12 transmission electron microscope equipped with a CCD camera.

### 2.7 Western blotting analysis

For Western blotting (WB), a standard protocol was performed.^31^ Proteins were extracted from lysed exosomes. A total of 50 μg of protein sample were separated by 10% SDS‐polyacrylamide gels, and electroblotted to polyvinylidene difluoride membranes (Millipore, Billerica, MA, USA). After sealed off by 5% (weight/volume) nonfat milk dissolved in a standard tris‐buffered saline solution with tween 20 (TBST), membranes were incubated at 4°C overnight with the first antibody. Subsequently, corresponding secondary antibodies, including Horseradish peroxidase (HRP)‐conjugated anti‐rabbit (CST, Danvers, MA, USA) and HRP‐conjugated anti‐mouse (GE Healthcare, Amersham, UK), were utilized on membranes for an hour at 37°C. Lastly, the detection was achieved on the C hemiDoc XRS + Molecular Imager (Bio‐Rad) with Luminata™ Crescendo Western HRP Substrate (Millipore, Billerica, MA, USA). The antibodies used for the WB detection were: anti‐ HSP70 (CST, Danvers, MA, USA), CD63 (OriGene Technologies, Rockville, MD, USA), Tsg101 (Proteintech, Wuhan, China), LMP1 (DAKO Lifetech, Glostrup, Denmark), and CYPA (Proteintech, Wuhan, China). GAPDH (Proteintech, Wuhan, China) was used as a protein loading control.

### 2.8 Enzyme‐linked immunosorbent assay

The anti‐CYPA antibody was diluted in phosphate‐buffered saline (PBS) to a final concentration of 0.5 μg/mL and coated onto a 96‐well microtiter plate (CUSABIO, Wuhan, China). Human sera diluted at 1:1 ratio were incubated in the antibody‐coated wells. HRP‐conjugated goat anti‐human IgG (Bioss, Beijing, China) and TMB (Hualen, Shanghai, China) were used as detection reagents. The optical density (OD) value was measured at 450 nm using an automated plate reader (Beckman, USA). For exosomal samples treatment, the collected exosomes were lysed from human sera were suspended in 200 μl RIPA buffer (including 1% PMSF) and then diluted at 1:20 ratio to incubate in a 96‐well microtiter plate coated with CYPA antibody.

For the quantitative determination of EBV‐VCA‐IgA in human sera, commercial immunoassay kits (Beier Bioengineering, Beijing, China) were purchased. The Enzyme‐linked immunosorbent assay (ELISA) assay was performed according to the manufacturer's instructions.

### 2.9 Quantitative polymerase chain reaction

Total RNA was extracted using the TRIzol Reagent (Vazyme, Nanjing, China) and cDNA samples were synthesized using a reverse transcription (RT) Kit (Transgen Biotech, Beijing, China). Real‐time quantitative polymerase chain reaction (qPCR) was performed in triplicate with SYBR Green PCR Kits (TransGen Biotech, Beijing, China), using β‐actin (Actin) as an internal control to normalize the expression of CYPA. The fold changes were calculated by the method of 2‐△△Ct method and expressed as a fold change. The following primers were used for real‐time amplification: *CYPA* (Forward 5′‐CAAGGTCCCAAAGACAGCAGA‐3′ and Reverse 5′‐AAGATGCCAGGACCCGTATGC‐3′); *LMP1* (Forward 5′ TGAACACCACCACGATGACT 3′ and Reverse 5′ GTGCGCCTAGGTTTTGAGAG 3′); *β‐actin* (Forward 5′‐TAGTTGCGTTACACCCTTTCTTG‐3 ′ and Reverse 5 ′ ‐TGCTGTCACCTTCACCGTTC‐3 ′).

### 2.10 Statistical analysis

Statistical analysis was performed using MedCalc (MedCalc for Windows, version 15.6.1.0, www.medcalc.be). The OD value analysis of CYPA was using a Student's *t* test when the data were normally distributed or the Mann‐Whitney U test. Receiver operating characteristics (ROC) analyses were performed to determine their potential diagnostic performance for differentiating NC and NPC. Areas under the ROC curve (AUC) with 95% confidence intervals (95% CI) were calculated. The cutoff values were determined according to Youden index and differences in diagnostic performance were analyzed by comparing the ROC curves of MedCalc software (Version 15.6). All statistical tests were 2 sided, and error bars in the graphs represent standard deviations. Graphics were analyzed using the GraphPad Prism program software (Prism 5.0). Significant differences: *P < 0.05; **P < 0.01; ***P < 0.001.

## 3 RESULTS

### 3.1 The verification of CYPA upregulation in NPC sera and tissues

**Figure 1:**
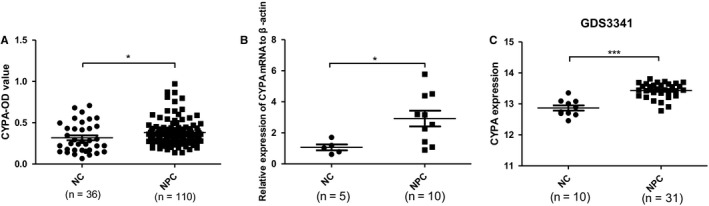
The expression levels of cyclophilin A (CYPA) in nasopharyngeal carcinoma (NPC) sera and tissues. A, CYPA levels in the whole sera detected by enzyme‐linked immunosorbent assay. Data were represented as mean ± sd of three independent experiments. B, The mRNA levels of CYPA in NPC tissues (n = 10) confirmed by quantitative polymerase chain reaction, and compared with normal cases (NC) tissues (n = 5). C, Gene microarray analysis from the Gene Expression Omnibus database (GDS3341) to compare CYPA expression between NPC patients (n = 31) and NC (n = 10). *P < 0.05, **P < 0.01, ***P < 0.001, as comparison to NC group

CYPA was previously found to be overexpressed in different TNM stages of NPC tissues by proteomics and immunohistochemical (IHC) analyses in our laboratory.^17^ Here the expressions of CYPA in NPC at protein level in sera and mRNA level in NPC tissues were evaluated by ELISA and qPCR, respectively. The CYPA protein in the whole sera from NPC patients was subjected to ELISA, and serum CYPA protein was elevated significantly in NPC patients compared with NC (*P* < 0.05, Figure 1A). Figure 1B showed the CYPA upregulation at mRNA level in NPC tissues (n = 10) (*P*< 0.05). We also analyzed the gene microarray data from the GEO database (GDS3341). As shown in Figure 1C, the CYPA gene expression in NPC tissues was significantly higher than that in NC ( *P*< 0.0001).

### 3.2 The high level of CYPA in serum exosomes of NPC

**Figure 2:**
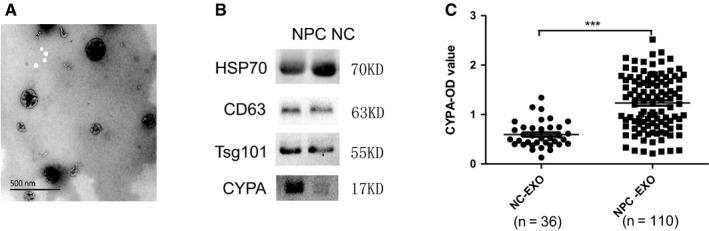
The expression levels of cyclophilin A (CYPA) in nasopharyngeal carcinoma serum exosomes. A, Electron microscopy identification of the extracellular vesicles of exosomes from human sera. B, Western blotting analysis of exosomal special markers (HSP70, CD63, and Tsg101) and CYPA expression in serum exosomes. C, Exosomal CYPA detection by enzyme‐linked immunosorbent assay. Data were represented as mean ± sd of three independent experiments. *P < 0.05, **P < 0.01, ***P < 0.001, as comparison to normal cases group

The alteration in exosomes can reflect the pathogenic condition of body. In this study, in order to know whether CYPA could be conveyed into the sera of NPC patients by exosomes, exosomes were extracted from NPC serum samples. The serum exosomes were characterized by electron microscopy (Figure 2A). As shown in Figure 2A, the vesicles were presented to be nano‐sized ranging from 30 to 200 nm in diameter and with round doubled disk structure. The exosomal markers (HSP70, CD63, and Tsg101) and CYPA in exosomes were assayed by Western blotting (Figure 2B), showing the high level of exosomal CYPA protein. The levels of exosomal CYPA in the samples were detected by ELISA, showing that exosomal CYPA existed at a significant higher level in NPC exosomes than that in NC (*P*< 0.0001) (Figure 2C).

### 3.3 Comparative analysis of diagnostic value of CYPA from whole sera and serum exosomes

**Figure 3:**
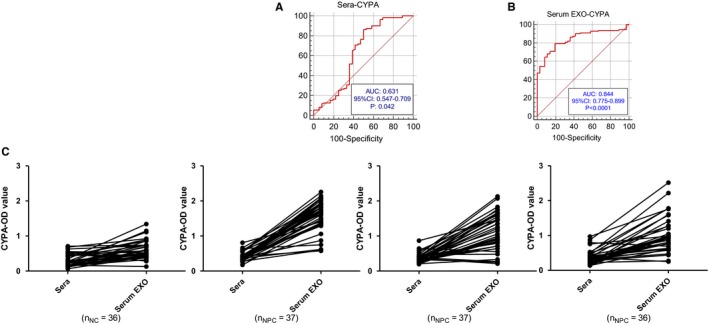
Diagnostic significance analysis of cyclophilin A (CYPA) levels in whole sera and serum exosomes. A, Receiver operating characteristics (ROC) curve analysis of CYPA from the whole sera. B, ROC curve analysis of CYPA from serum exosomes. C, Comparison analysis between sera CYPA and exosomal CYPA levels from the same individual samples. Data were represented as mean ± sd of three independent experiments. *P < 0.05, **P < 0.01, ***P < 0.001, as comparison to normal cases group. Sera stands for serum CYPA level; Serum EXO stands for serum exosomes

To confirm CYPA as a potential biomarker, ROC curves were utilized to analyze the diagnostic values of sera and exosomal CYPA on the basis of the above data. As shown in Figure 3A, B, the ROC curves discriminated between NC and NPC, with the area under the ROC curve (AUCs) being of 0.631 (CYPA detected from whole sera; 95% CI: 0.547‐0.709; *P* = 0.042; The cutoff value of human sera: 0.248) and 0.844 (CYPA from serum exosomes; 95% CI: 0.775‐0.899; *P* < 0.0001; The cutoff value of serum exosomes: 0.7434), respectively. As AUCs are an evaluation criterion, exosomal CYPA presented a high and serum CYPA presented a moderate diagnostic significance. It is noticeable that only 2.5% of serum amount for each sample was used in the exosomal CYPA detection by ELISA, compared with the detection in whole sera. Nevertheless, the detection values of exosomal CYPA were relatively higher than that of serum CYPA from the same sera samples (Figure 3C).

In addition, in consideration of the relatively low positive rate of EBV‐VCA‐IgA in NPC patients, we evaluated the combination of these two markers in the diagnosis of NPC. As it is shown in Tables 1 and 2, the results suggested that serum CYPA could make up for the default of utilizing serum EBV‐VCA‐IgA antibody alone in the diagnosis of NPC alone. For those samples with EBV‐VCA‐IgA negative, more than 80% of them showed that exosomal and serum CYPA were positive, and exosomal CYPA had a higher specificity with a false‐positive ratio of 6/36.

**Table 1:** Detection of EBV‐VCA‐IgA in human sera

	EBV(+)	EBV(‐)
NPC	68.2% (75/110)	31.8% (35/110)
NC	0 (0/36)	100% (36/36)

EBV, Epstein‐Barr virus; EXO, exosomes; NC, normal cases; NPC, nasopharyngeal carcinoma.

**Table 2:** Combinative analysis of EBV‐VCA‐IgA and CYPA level in human sera and exosomes

CYPA(+)	EBV(+)		EBV(‐)	
	Sera	Serum EXO	Sera	Serum EXO
NPC	84.0% (63/75)	78.7% (59/75)	91.4% (32/35)	80.0% (28/35)
NC	0	0	47.2% (17/36)	16.7% (6/36)

CYPA, cyclophilin A; EBV, Epstein‐Barr virus; EXO, exosomes; NC, normal cases; NPC, nasopharyngeal carcinoma.

### 3.4 The relationship of serum exosomal CYPA and LMP1 levels in NPC

**Figure 4:**
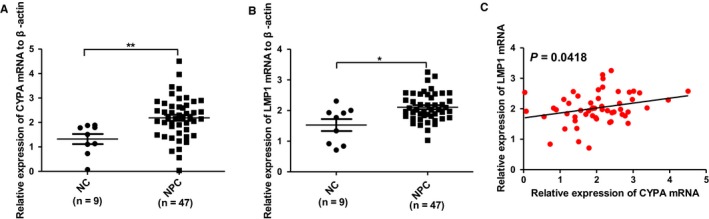
The relationship between exosomal cyclophilin A (CYPA) and latent membrane protein 1 (LMP1) in nasopharyngeal carcinoma (NPC). A, Exosomal CYPA levels in NPC sera samples detected by quantitative polymerase chain reaction. B, Exosomal LMP1 levels in the same sera samples as in (A). C, Correlation analysis between exosomal CYPA and LMP1 levels. Data were represented as mean ± sd of three independent experiments. *P < 0.05, **P < 0.01

The EBV oncoprotein LMP1 is able to be secreted into exosomes from EBV‐infected cells. We detected both the CYPA and LMP1 levels simultaneously in part of the NPC serum exosomes samples by qPCR (Figure 4A, B). The correlation analysis showed that there is a significantly positive correlation between exosomal CYPA and LMP1 (Figure 4C).

### 3.5 Exosomal CYPA expression in EBV‐positive cell lines

**Figure 5:**
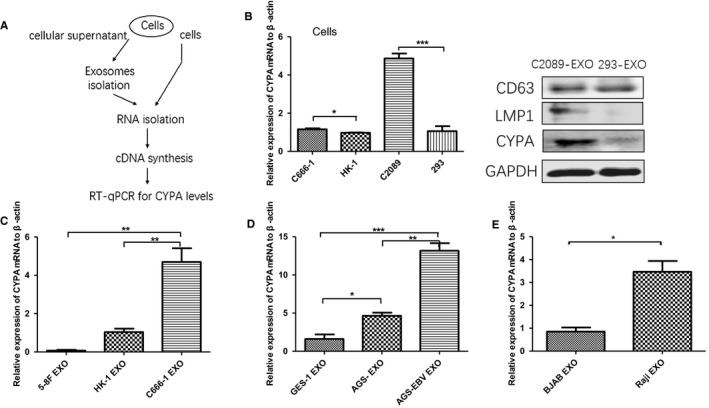
The expression of exosomal cyclophilin A (CYPA) in Epstein‐Barr virus (EBV) positive and negative cell lines. A, Operation steps of quantitative polymerase chain reaction (qPCR) with cells and exosomes derived from cells. B, qPCR analysis of CYPA in the EBV‐positive cells and EBV‐negative cells (C666‐1 VS HK‐1, C2089 VS 293) and Western blotting analysis of C2089 and 293. C, qPCR analysis of exosomal CYPA from EBV‐positive cells of nasopharyngeal carcinoma (NPC) (C666‐1) compared with that from EBV‐negative cells of NPC (5‐8F and HK‐1). D, qPCR analysis of exosomal CYPA from EBV‐positive cells of gastric carcinoma (AGS‐EBV) compared with that from EBV‐negative cells of gastric carcinoma (AGS‐). E, qPCR analysis of exosomal CYPA from EBV‐positive cells of lymphoma (Raji) compared with that from EBV‐negative cells of lymphoma (BJAB). Data were represented as mean ± sd of several individual experiments. *P < 0.05, **P < 0.01, ***P < 0.001

To further validate the correlation of exosomal CYPA with EBV infection, EBV‐ positive and negative cancer cell lines were used for CYPA detection as described in Figure 5A. According to Figure 5B, in EBV‐positive cells, CYPA had higher transcriptional levels than that in EBV‐negative cells. As a corresponding result, all the EBV‐positive cells, including EBV‐genome transfected cells (C2089), NPC cells (C666‐1), gastric cancer cells (AGS‐EBV), and lymphoma cells (Raji), exhibited higher levels of exosomal CYPA when compared with EBV‐negative cells (293, HK‐1, 5‐8F, AGS‐, BJAB) (Figure 5B‐E).

## 4 DISCUSSION

NPC is curable when diagnosed early and its 5‐year survival could be reached as much as 90%. However, most patients has been identified at advanced stages with a much lower 5‐year survival (<50%) clinically.^34^ Therefore, early diagnosis of NPC is crucial and there is a need for novel biomarkers to assist early detection.

EBV is closely linked to NPC, herein, the detection of EBV‐related factors has been developed and utilized in NPC diagnosis.^35^ EBV DNA by liquid biopsy has been verified as an outstanding tool in the early diagnosis EBV‐associated NPC.^36^ Several EBV antigens along with their specific antibodies are produced among the process of NPC development, including viral capsid antigen (VCA‐IgA) and intracellular early antigen (EA). These two indexes have been combined in an investigation for clinical diagnosis and monitoring.^34^ VCA‐IgA is more usually used. The sensitivity of VCA‐IgA is not high enough (about 60%‐70%) to meet the needs for clinic.^34^ Similarly, the detection rate of VCA‐IgA in our research was only 68.2% (Table 1), with about 30% of patients missing diagnosis, probably due to the immunocompromise with a cancer burden.^37^ In view of this, a protein in sera is not directly limited by immune status, could be a potential solution. Based on our study, circulating exosomal CYPA is proposed (Table 2).

Accumulating studies have disclosed significant properties and functions about CYPA.^10^ From our laboratory, Yang J, et al first reported the upregulation of CYPA in NPC in early‐stage NPC,^17^ implying that CYPA could be used for NPC early diagnosis. However, how to utilize it for the diagnosis remained to be explored. In this study, through a comparative analysis of CYPA protein expression, combined with the detection of VCA‐IgA, we concluded that exosomal CYPA is a promising biomarker for EBV‐associated NPC. The combination of exosomal CYPA and VCA‐IgA would increase the diagnosis sensitivity and specificity. This is also a noninvasive detection of liquid biopsy.

CYPA was primarily regarded as a cytosolic protein that binding with immunosuppressive drug cyclosporine A.^10^ While in recent years, CYPA has been proven to function outside of cells. For example, once tissues are injured, CYPA can be secreted from cells to recruit inflammatory cells.^8^, ^10^ It was the property of extracellular secretion of CYPA that suggested us to detect it in NPC sera.

Exosomes functions as a carrier of biological molecules, including proteins, lipids, DNA, and RNA, to facilitate intercellular communication.^38^ Due to the protection of exosomes, the molecules in it become much more stable in body liquid. There are limited reports about the correlation between CYPA and exosomes. Wang J‐J, et al verified that exosomes secreted from macrophages infected with *mycobacterium avium* contained upregulated CYPA.^39^ In prostate cancer cell, CYPA was present as a housekeeping protein in exosomes.^40^ In this study, we also proved that CYPA present in serum exosomes from NPC patients. Based on our study, CYPA not only could be drained into peripheral blood, but also could be conveyed and enriched into serum exosomes.

Though exosomes are nanoparticles in size, they possess the ability to permit the enrichment of proteins, especially in sera [26]. In our experiments, 100 μl of serum per sample was used for the extraction of exosomes and finally diluted in 200 μl of RIPA lysate. Furthermore, only 5 μl of diluted exosomal lysate, together with 95 μl of sample diluent, was subject to the ELISA. While, a total of 100 μl sera were applied for the ELISA in the detection of whole sera, with a lower CYPA level than that in the exosomes detection. This indicated that exosomal CYPA had a much more efficient value in NPC diagnosis (Figure 3A, B). Nevertheless, the extraction of exosomes from cell culture supernatant is still a problem due to the low production and, real‐time qPCR has been recommended to be an alternative detection of exosmal protein from cell culture rather than Western blotting.^41^

In addition, our results suggested that serum CYPA could make up for the default of utilizing EBV‐VCA‐IgA in the diagnosis of NPC alone. Among the 30% of NPC samples with VCA‐IgA negative, the rate that CYPA levels could be detected up to around 80%. Hence, we recommended a detection of exosomal CYPA for those suspected NPC patients with negative EBV‐VCA‐IgA. CYPA might not be ideal to act as a standalone indicator for NPC screening, which is probably due to the multiple functions of CYPA in normal physiological processes. Thus, the elevated level of CYPA in sera is not the only specific reaction in EBV‐associated NPC.

EBV is the most important etiological factor for NPC development and progression. EBV infection is an early event in NPC development because CYPA is also upregulated from the early stage of NPC, we tried to find a relationship between CYPA expression and EBV infection. LMP1 is the viral oncoprotein and was previously found to be packaged into host exosomes. We detected the expression levels of both in the same exosome samples, and the result showed they had a positive relationship (Figure 4C).

We also detected their expression in exosomes from EBV‐ positive and negative cell lines by performing RT‐qPCR (Figure 5A). The result displayed that exosomal CYPA from EBV‐positive cell line C2089 was higher than that from EBV‐negative 293 cell line at protein level (Figure 5B). In the detection of exosomes derived from cell lines of several EBV‐associated tumors including NPC, gastric carcinoma, and lymphadenoma, the results revealed similar patterns (Figure 5B‐E). The results imply that exosomal CYPA level is related to EBV infection. Whereas, elevated high level of CYPA may also be present in these EBV‐associated tumors. Therefore, in clinical practice, comprehensive factors including clinical symptoms, results from pathological and serological examination should be taken into consideration in diagnosis.

All in all, this study demonstrated that circulating exosomal CYPA is a novel promising biomarker of NPC. Clinically, a combination of exosomal CYPA and EBV‐VCA‐IgA would increase the accuracy of diagnosis, especially when EBV‐VCA‐IgA is negative. The enrichment of CYPA in serum exosomes discloses its practical significance in diagnosis by liquid biopsy.

## CONFLICT OF INTEREST

No conflict of interest to declare related to this study.

## AUTHORS' CONTRIBUTIONS

LJH conceived the experiments, LLZ and ZLL designed the experiments and performed the experiments, interpreted the data; TJY and ZJH provide assistance with preparing and conducting the serum samples. YJ, XSY, ZJ, and LGY collected and analyzed the data. LJH and LLZ drafted the manuscript and wrote the paper; YJ, LJH, and LGY revised critically the manuscript. All authors read and approved the final manuscript.

## ETHICS APPROVAL AND CONSENT TO PARTICIPATE

All samples were collected with informed consent and the experiments were approved by the institutional ethics committee of Central South University.

## DATA AVAILABILITY STATEMENT

Literature collection was performed using PubMed. Statistical analyses were executed by MedCalc software (Version 15.6) and GraphPad (Prism 5.0). Raw and processed data are stored in corresponding author and are available upon request. The information of the gene microarray (GDS3341) were retrieved from GEO database (https://www.ncbi.nlm.nih.gov/geo/).

## References

[1] Lee AW , Ma BB , Ng WT , Chan AT . Management of nasopharyngeal carcinoma: Current practice and future perspective. J Clin Oncol. 2015;33(29):3356‐3364. 26351355 10.1200/JCO.2015.60.9347

[2] Tang L‐L , Chen W‐Q , Xue W‐Q , et al. Global trends in incidence and mortality of nasopharyngeal carcinoma. Cancer Lett. 2016;374(1):22. 26828135 10.1016/j.canlet.2016.01.040

[3] Caves EA , Butch RM , Cook SA , et al. Latent membrane protein 1 is a novel determinant of Epstein‐Barr virus genome persistence and reactivation. Msphere. 2017;2(6):e00453‐e517. 29134204 10.1128/mSphereDirect.00453-17 PMC5677982

[4] Wei WI , Sham JS . Nasopharyngeal carcinoma. Lancet. 2005;365(9476):2041. 15950718 10.1016/S0140-6736(05)66698-6

[5] Wang W‐Y , Lin T‐Y , Twu C‐W , et al. Long‐term clinical outcome in nasopharyngeal carcinoma patients with post‐radiation persistently detectable plasma EBV DNA. Oncotarget. 2016;7(27):42608‐42616. 27191654 10.18632/oncotarget.9323 PMC5173160

[6] Gurtsevitch VE , Senyuta NB , Ignatova AV , et al. Epstein‐Barr virus biomarkers for nasopharyngeal carcinoma in non‐endemic regions. J Gen Virol. 2017;98(8). 2118‐2127. 28786806 10.1099/jgv.0.000889

[7] Wang LW , Jiang S , Gewurz BE . Epstein‐Barr Virus LMP1 mediated oncogenicity. J Virol. 2017;91(21):e01718‐e1816. 28835489 10.1128/JVI.01718-16 PMC5640852

[8] Nigro P , Pompilio G , Capogrossi MC . Cyclophilin A: a key player for human disease. Cell Death Dis. 2013;4(10):e888. 24176846 10.1038/cddis.2013.410 PMC3920964

[9] Camilloni C , Sahakyan AB , Holliday MJ , et al. Cyclophilin A catalyzes proline isomerization by an electrostatic handle mechanism. Proc Natl Acad Sci USA. 2014;111(28):10203‐10208. 24982184 10.1073/pnas.1404220111 PMC4104850

[10] Obchoei S , Wongkhan S , Wongkham C , Li M , Yao Q , Chen C . Cyclophilin A: potential functions and therapeutic target for human cancer. Med Sci Monit. 2009;15(11):RA221. 19865066

[11] Satoh K . Cyclophilin A in cardiovascular homeostasis and diseases. Tohoku J Exp Med. 2015;235(1):1‐15. 25743766 10.1620/tjem.235.1

[12] Liu MC , Lee YW , Lee PT , et al. Cyclophilin A is associated with peripheral artery disease and chronic kidney disease in geriatrics: The Tianliao Old People (TOP) Study. J Am Coll Cardiol. 2015;65(17):9937. 10.1038/srep09937 PMC4408976 25909510

[13] Seizer P , Gawaz M , May AE . Cyclophilin A and EMMPRIN (CD147) in cardiovascular diseases. Cardiovasc Res. 2014;102(1):17. 24518139 10.1093/cvr/cvu035

[14] Saleh T , Jankowski W , Sriram G , et al. Cyclophilin A promotes cell migration via the Abl‐Crk signaling pathway. Nat Chem Biol. 2016;12(2):117‐123. 26656091 10.1038/nchembio.1981 PMC4718742

[15] Zhu DI , Wang Z , Zhao J‐J , et al. The Cyclophilin A‐CD147 complex promotes the proliferation and homing of multiple myeloma cells. Nat Med. 2015;21(6):572. 26005854 10.1038/nm.3867 PMC4567046

[16] Chen C , Li M , Yang H , Chai H , Fisher W , Yao Q . Roles of thymosins in cancers and other organ systems. World J Surg. 2005;29(3):264. 15706436 10.1007/s00268-004-7817-2

[17] Yang J , Zhou M , Zhao R , et al. Identification of candidate biomarkers for the early detection of nasopharyngeal carcinoma by quantitative proteomic analysis. J Proteomics. 2014;109:162‐175. 24998431 10.1016/j.jprot.2014.06.025

[18] Zhang X , Yuan X , Shi H , Wu L , Qian H , Xu W . Exosomes in cancer: small particle, big player. J Hematol Oncol. 2015;8(1):83. 26156517 10.1186/s13045-015-0181-x PMC4496882

[19] Liu L , Quan Z , Yan X , Zuo L , Zhu F , Lu J . Extracellular vesicles: novel vehicles in herpesvirus infection. Virol Sin. 2017;32(5):349‐356. 29116589 10.1007/s12250-017-4073-9 PMC6704204

[20] Kamerkar S , LeBleu VS , Sugimoto H , et al. Exosomes facilitate therapeutic targeting of oncogenic KRAS in pancreatic cancer. Nature. 2017;546(7659):498. 28607485 10.1038/nature22341 PMC5538883

[21] Qu LE , Ding J , Chen C , et al. Exosome‐transmitted lncARSR promotes sunitinib resistance in renal cancer by acting as a competing endogenous RNA. Cancer Cell. 2016;29(5):653. 27117758 10.1016/j.ccell.2016.03.004

[22] Zhang H , Deng T , Liu R , et al. Exosome‐delivered EGFR regulates liver microenvironment to promote gastric cancer liver metastasis. Nat Commun. 2017;8:15016. 28393839 10.1038/ncomms15016 PMC5394240

[23] Boyiadzis M , Whiteside TL . The emerging roles of tumor‐derived exosomes in hematological malignancies. Leukemia. 2017 31(6):1259‐1268. 28321122 10.1038/leu.2017.91

[24] Yoshizaki T , Kondo S , Wakisaka N , et al. Pathogenic role of Epstein‐Barr virus latent membrane protein‐1 in the development of nasopharyngeal carcinoma. Cancer Lett. 2013;337(1):1‐7. 23689138 10.1016/j.canlet.2013.05.018

[25] Aga M , Bentz GL , Raffa S , et al. Exosomal HIF1α supports invasive potential of nasopharyngeal carcinoma‐associated LMP1‐positive exosomes. Oncogene. 2014;33(37):4613. 24662828 10.1038/onc.2014.66 PMC4162459

[26] Hurwitz SN , Nkosi D , Conlon MM , et al. CD63 regulates Epstein‐Barr virus LMP1 exosomal packaging, enhancement of vesicle production, and non‐canonical NF‐κB signaling. J Virol. 2016;91(5). e02251‐e2316. 10.1128/JVI.02251-16 PMC5309960 27974566

[27] Huang DP , Ho J , Poon YF , et al. Establishment of a cell line (NPC/HK1) from a differentiated squamous carcinoma of the nasopharynx. Int J Cancer. 1980;26(2):127‐132. 6259064 10.1002/ijc.2910260202

[28] Cai L , Ye Y , Jiang Q , et al. Epstein‐Barr virus‐encoded microRNA BART1 induces tumour metastasis by regulating PTEN‐dependent pathways in nasopharyngeal carcinoma. Nat Commun. 2015;6:7353. 26135619 10.1038/ncomms8353 PMC4507016

[29] Cheung ST , Huang DP , Hui A , et al. Nasopharyngeal carcinoma cell line (C666–1) consistently harbouring Epstein‐Barr virus. Int J Cancer. 1999;83(1):121‐126. 10449618 10.1002/(sici)1097-0215(19990924)83:1<121::aid-ijc21>3.0.co;2-f

[30] Zuo L , Yu H , Liu L , et al. The copy number of Epstein‐Barr virus latent genome correlates with the oncogenicity by the activation level of LMP1 and NF‐κB. Oncotarget. 2015;6(38):41033‐41044. 26517512 10.18632/oncotarget.5708 PMC4747387

[31] Lu JH , Tang YL , Yu HB , et al. Epstein‐Barr virus facilitates the malignant potential of immortalized epithelial cells: from latent genome to viral production and maintenance. Lab Invest. 2010;90(2):196‐209. 19997065 10.1038/labinvest.2009.130

[32] Delecluse HJ , Hilsendegen T , Pich D , Zeidler R , Hammerschmidt W . Propagation and recovery of intact, infectious Epstein‐Barr Virus from prokaryotic to human cells. Proc Natl Acad Sci USA. 1998;95(14):8245‐8250. 9653172 10.1073/pnas.95.14.8245 PMC20961

[33] Yu H , Lu J , Zuo L , et al. Epstein‐Barr virus downregulates microRNA 203 through the oncoprotein latent membrane protein 1: a contribution to increased tumor incidence in epithelial cells. J Virol. 2012;86(6):3088. 22205737 10.1128/JVI.05901-11 PMC3302296

[34] Coghill AE , Hsu W‐L , Pfeiffer RM , et al. Epstein‐Barr virus serology as a potential screening marker for nasopharyngeal carcinoma among high‐risk individuals from multiplex families in Taiwan. Cancer Epidemiol Biomarkers Prev. 2014;23(7):1213. 24769890 10.1158/1055-9965.EPI-13-1262 PMC4082438

[35] Raab‐Traub N . Nasopharyngeal carcinoma: An evolving role for the Epstein‐Barr Virus. Curr Top Microbiol Immunol. 2015;390(Pt 1):339‐363. 26424653 10.1007/978-3-319-22822-8_14

[36] Chan K , Chu S , Lo Y . Ambient temperature and screening for nasopharyngeal cancer. N Engl J Med. 2018;378(10):962. 29514031 10.1056/NEJMc1800433

[37] Gao L , Wang L , Dai T , et al. Tumor‐derived exosomes antagonize innate antiviral immunity. Nat Immunol. 2018 19(3):233‐245. 29358709 10.1038/s41590-017-0043-5

[38] Greening DW , Gopal SK , Xu R , Simpson RJ , Chen W . Exosomes and their roles in immune regulation and cancer. Semin Cell Dev Biol. 2015;40:72. 25724562 10.1016/j.semcdb.2015.02.009

[39] Wang JJ , Chen C , Xie PF , Pan Y , Tan YH , Tang LJ . Proteomic analysis and immune properties of exosomes released by macrophages infected with Mycobacterium avium. Microbes Infect. 2014;16(4):283‐291. 24355714 10.1016/j.micinf.2013.12.001

[40] Webber J , Stone TC , Katilius E , et al. Proteomics analysis of cancer exosomes using a novel modified aptamer‐based array (SOMAscan™) platform. Mol Cell Proteomics. 2014;13(4):1050. 24505114 10.1074/mcp.M113.032136 PMC3977183

[41] Endzeliņš E , Berger A , Melne V , et al. Detection of circulating miRNAs: comparative analysis of extracellular vesicle‐incorporated miRNAs and cell‐free miRNAs in whole plasma of prostate cancer patients. BMC Cancer. 2017;17(1):730. 29121858 10.1186/s12885-017-3737-z PMC5679326

